# Diagnostic Efficacy of Ultrasound-Based International Ovarian Tumor Analysis Simple Rules and Assessment of the Different Neoplasias in the Adnexa Model in Malignancy Prediction Among Women With Adnexal Masses: A Systematic Review

**DOI:** 10.7759/cureus.67365

**Published:** 2024-08-21

**Authors:** Shweta V Suryawanshi, Kanchan S Dwidmuthe, Snehal Savalkar, Anuja Bhalerao

**Affiliations:** 1 Department of Obstetrics and Gynaecology, N.K.P. Salve Institute of Medical Sciences and Research Centre, Nagpur, IND; 2 Department of Surgery, Government Medical Hospital, Satara, IND

**Keywords:** diagnostic efficacy, ovarian tumors, adnexal masses, iota-adnex model, iota simple rules

## Abstract

Transvaginal ultrasonography (USG) is most commonly used before surgery to accurately diagnose benign and malignant ovarian masses for effective treatment, avoid unnecessary interventions, improve the prognosis of patients, and preserve fertility in patients with benign tumors. Therefore, the objective of the present systematic review was to assess the diagnostic efficacy of ultrasound-based International Ovarian Tumor Analysis (IOTA) Simple Rules (SR) and Assessment of Different NEoplasias in the adneXa (ADNEX) model in predicting malignancy among women with adnexal masses. A systematic literature search was carried out on electronic databases consisting of Science Direct, PubMed, and Google Scholar. The keywords utilized to perform the literature search and include relevant articles consisted of “Diagnostic Efficacy”, AND “Ultrasound-Based International Ovarian Tumor Analysis Simple Rules”, AND “International Ovarian Tumor Analysis ADNEX Model”, AND “Adnexal masses”, AND “Ovarian tumors”. Based on the selection criteria, a total of five studies were included. The study concluded that both the models showed high diagnostic efficacy for malignancy prediction; however, in comparison to the IOTA SR, the IOTA ADNEX model demonstrated good diagnostic efficacy.

## Introduction and background

Among gynaecological malignancies, with an average five-year survival rate of less than 50%, ovarian cancer has the poorest prognosis and greatest mortality rate [1,2]. Transvaginal ultrasonography (USG) is currently the most widely used, inexpensive, non-invasive imaging method for evaluating adnexal masses before surgery with the least risk and pain to the patient [3-5]. Furthermore, one of the most effective ways to assess adnexal masses in clinical practice is by the subjective evaluation of ultrasound results by specialists in gynaecological USG [5-10]. To provide effective treatment, avoid unnecessary interventions, maintain the fertility of patients, and improve the prognosis of patients, appropriate diagnosis through accurate staging of malignant and benign masses is essential [11-13].

Due to a lack of qualified or experienced examiners and their geographical unavailability, several ultrasound-based prediction models have been developed [14]. The International Ovarian Tumor Analysis (IOTA) group presented the consensus statement regarding the ultrasound characteristics of adnexal tumors in 2000 [15], and further diagnostic models, such as the simple rules (SRs) [16-18], and the Assessment of Different NEoplasias in the adneXa (ADNEX) [19] that were developed and validated subsequently. Although the SRs model was previously determined to be user-friendly, it was not appropriate for all adnexal masses [20]. Research has shown that the ADNEX model is a reliable predictor of malignant versus benign disease along with the stage of the disease [20]; however, there is limited evidence on the comparative analysis of SRs and the ADNEX model. Hence, the objective of the present systematic review is to assess the diagnostic efficacy of the ultrasound-based SR and ADNEX model in predicting malignancy among women with adnexal masses.

## Review

The guidelines Preferred Reporting Items for Systematic Reviews and Meta-analyses (PRISMA) were followed for this systematic review [21].

Data sources and search strategy

From 2019 to 2023, the literature search was conducted on electronic databases that involved Google Scholar, PubMed, and Science Direct. The keywords utilized to perform the literature search and include relevant articles consisted of “Diagnostic Efficacy”, AND “Ultrasound-Based International Ovarian Tumor Analysis Simple Rules”, AND “International Ovarian Tumor Analysis ADNEX Model”, AND “Adnexal masses”, AND “Ovarian tumors”.

Study screening and selection

For screening, the inclusion criteria consisted of studies involving women with adnexal masses and ovarian tumors in which the diagnostic efficacy of ultrasound-based IOTA SRs and ADNEX model was assessed for prediction of malignancy, studies published between 2019 and 2023, cross-sectional studies, observational studies, randomized controlled trials, retrospective observational studies, prospective cohort studies published in the English language, and studies with full-text availability. However, the exclusion criteria involved study designs that consisted of case reports, review articles, meta-analyses, commentaries, guidelines, editorials, book chapters, and letters to editors, studies not described in the English language, full-text non-availability, and providing insufficient information related to the context.

Two reviewers individually assessed each article to determine the applicability of the articles in the review. Firstly, the titles and abstracts of the articles were screened for the elimination of duplicate articles. Secondly, the articles that were selected were screened again to exclude articles that did not follow the eligibility criteria. Finally, the selected articles were screened based on the full text to determine eligibility. Any discrepancies or disagreements were settled through consensus and discussions.

Data extraction

The extracted data included the first author along with the year of publication, the study and location, the number of patients involved, the patients' age range, the type of tumor involved, the results demonstrating the diagnostic efficacy of both models, the conclusion, and the assessment of the quality of the studies. The data were extracted individually by the authors and were reviewed and compiled.

Quality assessment

For the included studies, the Mixed Methods Appraisal Tool (MMAT) was used for the assessment of methodological quality to evaluate studies that are qualitative, quantitative descriptive (cross-sectional), non-randomized, randomized controlled trials, and mixed methods [22,23]. The studies were graded as high, low, or of moderate quality based on the various parameters described in the tool.

Data synthesis

To summarize the results of the included studies, the critical narrative technique, which utilizes tables, text, and figures, to summarize and validate research findings was employed [22]. Higher methodological quality studies, incorporated study limitations, potential biases, and other factors were taken into consideration during the analysis of the findings, offering a critical perspective for the review. As relevant studies were limited in number, a meta-analyses approach or statistical synthesis was not suitable. Various research methodologies and outcome measures were considered in the included studies, leading to a significant degree of heterogeneity.

Results

**Figure 1:**
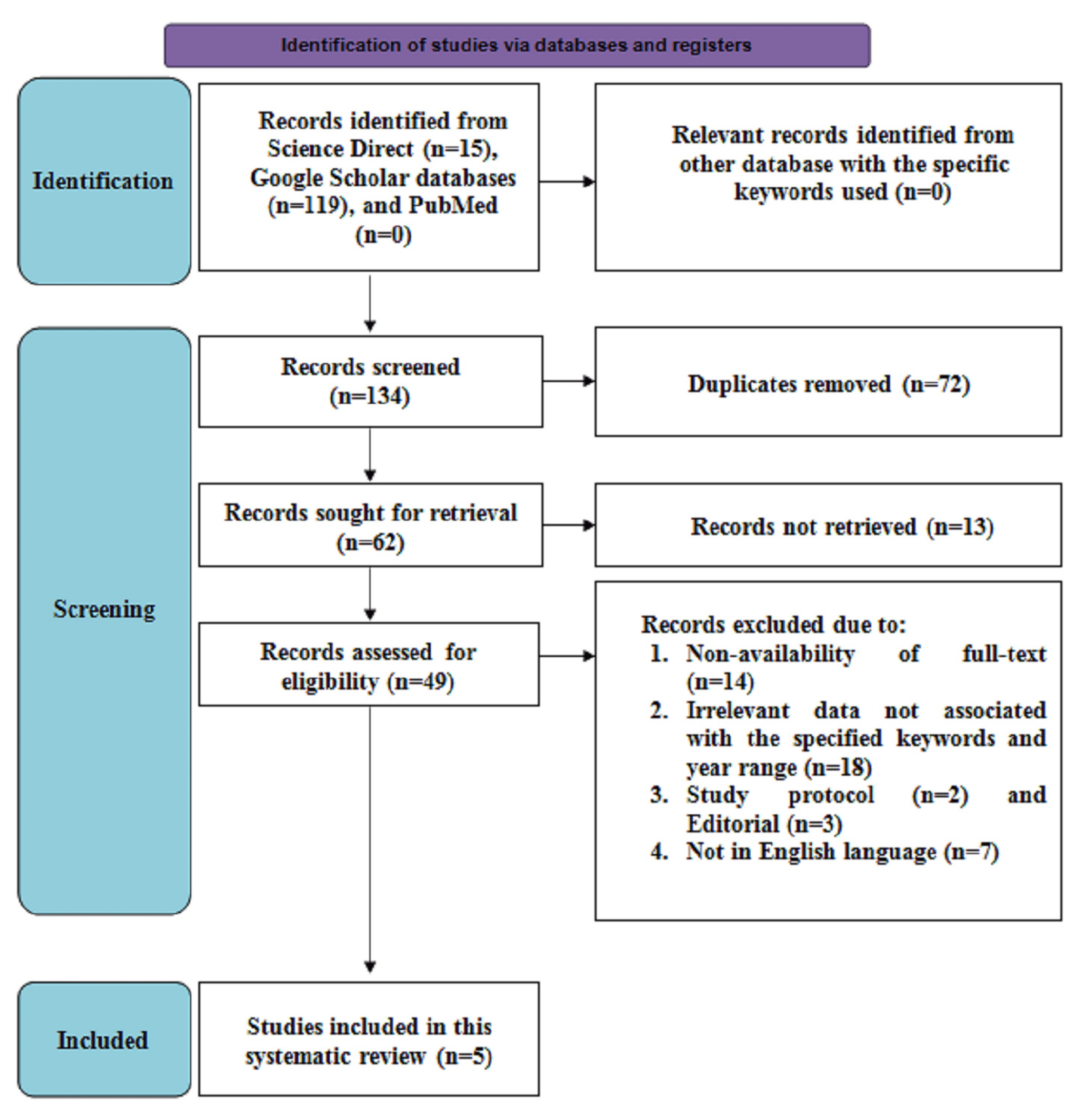
PRISMA flowchart illustrating the search strategy PRISMA = Preferred Reporting Items for Systematic Reviews and Meta-analyses

Figure 1 depicts the search strategy that is outlined in the PRISMA flow diagram. Initially, 134 articles were screened consisting of 119 studies from the Google Scholar database, 0 from the PubMed database, and 15 articles from the Science Direct database. The duplicate articles consisting of 72 in number were removed, after which 62 articles remained and were evaluated for retrieval, from which 13 articles were not retrieved. Following this, 49 articles were screened for eligibility from which 18 articles provided irrelevant data associated with the specified keywords, 14 articles reported non-availability of the full text, five were studies other than research or original articles (n=2 study protocols, n=3 editorials), and seven were not described in English language and therefore were excluded. Hence, a total of five studies consisting of observational studies, secondary analysis of cohort study, and retrospective and prospective studies describing comparative outcomes related to the diagnostic efficacy of ultrasound-based IOTA SRs and ADNEX model that were assessed for prediction of malignancy among women with adnexal masses were incorporated for this systematic review.

Table 1 describes the summary of the extracted data from the five included studies, consisting of the first author along with the year of publication, study design, location, sample size, patient age range, and type of tumor involved.

**Table 1:** Demographic parameters

Sr. No.	Author and year	Study design	Place	Sample size	Mean or Median Age (Age range) years	Type of tumor involved
1.	Qian et al. (2021) [13]	Observational cross-sectional study	China	486	Benign: 41 (31-51) years, malignant: 54 (42-63) years	Adnexal masses
2.	Rashmi et al. (2023) [20]	Prospective observational study	India	90	18–80 years	Ovarian tumors
3.	Pelayo et al. (2023) [24]	Retrospective observational study	Spain	122	51.4 (14-91) years	Adnexal masses
4.	Yoeli-Bik et al. (2023) [25]	Retrospective diagnostic study	United States of America (USA)	511	52.5±15.2 years	Adnexal masses
5.	Giourga et al. (2023) [26]	Diagnostic accuracy study	Greece	66	Benign: 46 years, malignant: 58 years	Adnexal masses

Furthermore, Table 2 presents the results with the diagnostic efficacy of both the models along with the conclusion and quality assessment.

**Table 2:** Diagnostic efficacy of both models SR = Simple Rules, ADNEX = Assessment of Different NEoplasias in the adneXa, PPV = Positive Predictive Value, NPV = Negative Predictive Value

Sr. No.	Author and year	Results								Conclusion	Quality assessment
		SR model				ADNEX model					
		Sensitivity	Specificity	PPV	NPV	Sensitivity	Specificity	PPV	NPV		
1.	Qian et al. (2021) [13]	93% (86%-97%)	86% (82%-89%)	87% (82%-91%)	92% (90.88%-95%)	93% (87%-97%)	76% (72%-81%)	80% (75%-84%)	92% (87%-95%)	Both the models demonstrated high diagnostic efficacy however, SR reported highest sensitivity and specificity in comparison to the ADNEX model.	High
2.	Rashmi et al. (2023) [20]	66.6%	91%	72%	95%	80%	92%	57%	87%	Both the models demonstrated high diagnostic efficacy however, ADNEX model reported highest sensitivity and specificity in comparison to the SR.	High
3.	Pelayo et al. (2023) [24]	66.7% (52.2%–81.2%)	89.2% (82.1%–96.3%)	72.0%	86.6%	95.1% (88.7%–100%)	74.1% (65.9%–82.3%)	65%	96.8%	Both the models demonstrated high diagnostic efficacy however, ADNEX model reported highest sensitivity and specificity in comparison to the SR.	High
4.	Yoeli-Bik et al. (2023) [25]	93.8% (86.2%-98.0%)	88.1% (84.7%-91.0%)	59.8% (50.8%-68.4%)	98.7% (97.0%-99.6%)	91.4% (83.0%-96.5%)	86.3% (82.7%-89.4%)	55.6% (46.8%-64.2%)	98.1% (96.2%-99.3%)	Both the models demonstrated high diagnostic efficacy however, SR reported highest sensitivity and specificity in comparison to the ADNEX model.	High
5.	Giourga et al. (2023) [26]	76.92%-92.00%	90.32%-96.88%	87.50%-95.24%	83.78%-94.44%	96.30%-100%	35.90%-79.49%	50.98%-77.14%	93.33%-100%	Both the models demonstrated high diagnostic efficacy however, ADNEX model reported highest sensitivity and specificity in comparison to the SR.	High

The present systematic review highlighted the diagnostic efficacy of IOTA SR and the ADNEX model for malignancy prediction among women with adnexal masses. The findings of the various studies demonstrated that both models reported high diagnostic efficacy. However, two studies stated the superiority of SR over the ADNEX model and three studies described more diagnostic efficacy of the ADNEX model in comparison to the SR. The sensitivity, specificity, positive predictive value (PPV), and negative predictive value (NPV) of the SR model ranged from 52.2% to 98.0%, 82.0% to 96.88%, 50% to 95.24%, and 83.78% to 99.6%, respectively. Meanwhile, for the ADNEX model, the values ranged from 80% to 100%, 35.90% to 92%, 46.8% to 84%, and 87% to 100%, respectively. Similarly, previous research found that the ADNEX model had better diagnostic performance, making it ideal for differentiating adnexal masses before surgery [6,19]. Additionally, when the ADNEX model and CA-125 were combined, the diagnostic efficacy was maximum for both malignant and benign tumor prediction. Moreover, magnetic resonance imaging is not required as the IOTA models have higher specificity in malignancy prediction [27], which is considered a huge advantage for developing regions.

For ensuring effective treatment, distinguishing between malignant and benign adnexal tumors is essential. The preoperative differentiation between both types of lesions needs a combined strategy, including the clinical examination, patient's medical history, tumor markers profile, and imaging. USG has the inherent advantages of being readily available, inexpensive, and free of radiation dangers. To recognize the specific pathology and distinguish between malignant and benign lesions, subjective evaluation using Doppler and ultrasound-based pattern recognition was used [28]. The ultrasound accurately characterizes the lesions if commenced using the IOTA standard terminology for planning appropriate management. For pre- and postmenopausal women, the IOTA-based prediction model outperformed the risk of ovarian malignancy algorithm (ROMA) in malignancy risk prediction [29]. 

There is always a tradeoff between sensitivity and specificity when comparing USG models, and balance is determined by several factors, including the complications that are related to the surgery in a patient with multiple comorbidities, infrastructure, the risk tolerance for missing cancer by the patient and the physician, approval of the insurance, and access to surgeons. Lowering specificity may result in more follow-ups related to MRI, more referrals, and more surgeries on benign tumors. In populations wherein the prevalence of malignant tumors is low, the challenge of balancing specificity and sensitivity in USG models is furthermore evident, which impacts PPVs and consequently increases false-positive outcomes. Before determining an individual cutoff, the patient and the physician must consider the risks and advantages in the context of the adnexal mass evaluation [30-33].

Strengths and limitations

The strength highlighted by the present study involved the comparative analysis between ultrasound-based IOTA-SR and IOTA ADNEX model concerning their diagnostic efficacy as the data regarding both parameters are scant. Moreover, considering the systematic review, all the studies included were of the highest quality demonstrating the efficacy and effectiveness of both the techniques. However, the systematic review described certain limitations, which involved, firstly, a small sample size included in the studies, no reporting of meta-analysis because of the heterogeneous nature in the methodological part of the studies included, and a small number of studies considered due to the selection criteria imposed. Additionally, research published in languages other than English was not included, which might have limited the number of relevant publications. Lastly, studies published in journals that are less indexed or published in databases other than those considered were excluded.

## Conclusions

The present study reported that the ultrasound-based IOTA SR and IOTA ADNEX model showed high diagnostic efficacy for malignancy prediction in women with adnexal masses. However, in comparison to the IOTA SR, the IOTA ADNEX model demonstrated good diagnostic efficacy. To facilitate standardized reporting, sonographic evaluation, and enhance consistency, these models were developed particularly for non-experts to reduce the complicated demonstration of masses into objective variables, minimizing the count of unclear reports, which frequently result in benign lesion surgeries.

## References

[1] Jayson GC, Kohn EC, Kitchener HC, Ledermann JA (2014). Ovarian cancer. Lancet.

[2] Siegel RL, Miller KD, Jemal A (2019). Cancer statistics, 2019. CA Cancer J Clin.

[3] Piovano E, Cavallero C, Fuso L (2024). Diagnostic accuracy and cost-effectiveness of different strategies to triage women with adnexal masses: a prospective study. Ultrasound Obstet Gynecol.

[4] Menon U, Gentry-Maharaj A, Hallett R (2009). Sensitivity and specificity of multimodal and ultrasound screening for ovarian cancer, and stage distribution of detected cancers: results of the prevalence screen of the UK Collaborative Trial of Ovarian Cancer Screening (UKCTOCS). Lancet Oncol.

[5] Fischerova D (2011). Ultrasound scanning of the pelvis and abdomen for staging of gynecological tumors: a review. Ultrasound Obstet Gynecol.

[6] Meys EM, Jeelof LS, Achten NM, Slangen BF, Lambrechts S, Kruitwagen RF, Van Gorp T (2017). Estimating risk of malignancy in adnexal masses: external validation of the ADNEX model and comparison with other frequently used ultrasound methods. Ultrasound Obstet Gynecol.

[7] Meys EM, Kaijser J, Kruitwagen RF (2016). Subjective assessment versus ultrasound models to diagnose ovarian cancer: a systematic review and meta-analysis. Eur J Cancer.

[8] Valentin L, Hagen B, Tingulstad S, Eik-Nes S (2001). Comparison of 'pattern recognition' and logistic regression models for discrimination between benign and malignant pelvic masses: a prospective cross validation. Ultrasound Obstet Gynecol.

[9] Van Gorp T, Veldman J, Van Calster B (2012). Subjective assessment by ultrasound is superior to the risk of malignancy index (RMI) or the risk of ovarian malignancy algorithm (ROMA) in discriminating benign from malignant adnexal masses. Eur J Cancer.

[10] Timmerman D, Schwärzler P, Collins WP (1999). Subjective assessment of adnexal masses with the use of ultrasonography: an analysis of interobserver variability and experience. Ultrasound Obstet Gynecol.

[11] Valentin L, Ameye L, Testa A (2024). Ultrasound characteristics of different types of adnexal malignancies. Gynecol Oncol.

[12] Di Legge A, Testa AC, Ameye L (2012). Lesion size affects diagnostic performance of IOTA logistic regression models, IOTA simple rules and risk of malignancy index in discriminating between benign and malignant adnexal masses. Ultrasound Obstet Gynecol.

[13] Qian L, Du Q, Jiang M, Yuan F, Chen H, Feng W (2021). Comparison of the diagnostic performances of ultrasound-based models for predicting malignancy in patients with adnexal masses. Front Oncol.

[14] Terzic M, Aimagambetova G, Norton M (2021). Scoring systems for the evaluation of adnexal masses nature: current knowledge and clinical applications. J Obstet Gynaecol.

[15] Timmerman D, Valentin L, Bourne TH, Collins WP, Verrelst H, Vergote I (2024). Terms, definitions and measurements to describe the sonographic features of adnexal tumors: a consensus opinion from the International Ovarian Tumor Analysis (IOTA) group. Ultrasound Obstet Gynecol.

[16] Timmerman D, Testa AC, Bourne T (2008). Simple ultrasound-based rules for the diagnosis of ovarian cancer. Ultrasound Obstet Gynecol.

[17] Timmerman D, Ameye L, Fischerova D (2010). Simple ultrasound rules to distinguish between benign and malignant adnexal masses before surgery: prospective validation by IOTA group. BMJ.

[18] Timmerman D, Van Calster B, Testa A (2016). Predicting the risk of malignancy in adnexal masses based on the simple rules from the International Ovarian Tumor Analysis Group. Am J Obstet Gynecol.

[19] Van Calster B, Van Hoorde K, Valentin L (2014). Evaluating the risk of ovarian cancer before surgery using the ADNEX model to differentiate between benign, borderline, early and advanced stage invasive, and secondary metastatic tumours: prospective multicentre diagnostic study. BMJ.

[20] Rashmi N, Singh S, Begum J, Sable MN (2023). Diagnostic performance of ultrasound-based International Ovarian Tumor Analysis simple rules and assessment of different neoplasias in the adnexa model for predicting malignancy in women with ovarian tumors: a prospective cohort study. Womens Health Rep (New Rochelle).

[21] Page MJ, McKenzie JE, Bossuyt PM (2021). The PRISMA 2020 statement: an updated guideline for reporting systematic reviews. BMJ.

[22] Garrington C, O'Shea S, Pope R (2024). Prevention and management of urinary incontinence, anal incontinence and pelvic organ prolapse in military women and female elite athletes. J Mil Veterans Health.

[23] Hong QN, Pluye P, Fàbregues S (2019). Improving the content validity of the mixed methods appraisal tool: a modified e-Delphi study. J Clin Epidemiol.

[24] Pelayo M, Pelayo-Delgado I, Sancho-Sauco J (2023). Comparison of ultrasound scores in differentiating between benign and malignant adnexal masses. Diagnostics (Basel).

[25] Yoeli-Bik R, Longman RE, Wroblewski K, Weigert M, Abramowicz JS, Lengyel E (2023). Diagnostic performance of ultrasonography-based risk models in differentiating between benign and malignant ovarian tumors in a US cohort. JAMA Netw Open.

[26] Giourga M, Pouliakis A, Vlastarakos P (2024). Evaluation of IOTA-ADNEX model and simple rules for identifying adnexal masses by operators with varying levels of expertise: a single-center diagnostic accuracy study. Ultrasound Int Open.

[27] Hiett AK, Sonek JD, Guy M, Reid TJ (2022). Performance of IOTA simple rules, simple rules risk assessment, ADNEX model and O-RADS in differentiating between benign and malignant adnexal lesions in North American women. Ultrasound Obstet Gynecol.

[28] Sokalska A, Timmerman D, Testa AC (2009). Diagnostic accuracy of transvaginal ultrasound examination for assigning a specific diagnosis to adnexal masses. Ultrasound Obstet Gynecol.

[29] Kaijser J, Bourne T, Valentin L (2013). Improving strategies for diagnosing ovarian cancer: a summary of the International Ovarian Tumor Analysis (IOTA) studies. Ultrasound Obstet Gynecol.

[30] Levine SM, Marciniuk DD (2022). Global impact of respiratory disease: what can we do, together, to make a difference?. Chest.

[31] Jurkovic D (2023). Conservative management of adnexal tumors: how to tell good from bad. Ultrasound Obstet Gynecol.

[32] Baumgarten DA (2022). O-RADS: good enough for everyday practice or a work in progress?. Radiol Imaging Cancer.

[33] Baumgarten DA (2022). A simplified approach to adnexal lesions may be enough. Radiology.

